# See better, treat better – cap-assisted single-operator pancreatoscopy for electrohydraulic lithotripsy of pancreatic duct stones

**DOI:** 10.1055/a-2948-3298

**Published:** 2026-09-10

**Authors:** Mark Ellrichmann, Nikolaus Schäfer, Franziska Halfmann, Hauke Hamel, Hinrich Fehrendt, Christian Meinhardt

**Affiliations:** 1 Department of Internal Medicine, Gastroenterology, Klinikum OldenburgUniversity Medical Center OldenburgOldenburgGermany

Chronic calcifying pancreatitis with obstructive pancreatic duct stones (Cambridge grade IV) remains a therapeutic challenge. 1​ Although endoscopic treatment is the preferred minimally invasive approach, impacted pancreatic duct stones often preclude complete ductal clearance. 2​ Single-operator pancreatoscopy (SOP) with electrohydraulic lithotripsy (EHL) has expanded therapeutic options 3​ ​4​ ; however, the precise intraductal orientation and targeting remain technically demanding.

A 44-year-old patient with a 20-year history of idiopathic chronic calcifying pancreatitis presented with recurrent epigastric pain radiating to the back after a 10-year symptom-free interval. Twelve weeks earlier, endoscopic retrograde cholangiopancreatography (ERCP) had demonstrated multiple obstructing pancreatic duct stones. Partial stone extraction and placement of a 10-Fr/10-cm pancreatic duct stent restored ductal drainage.

At repeat ERCP, pancreatography after stent removal revealed multiple intraparenchymal calcifications, irregular dilation of the main pancreatic duct (up to 8 mm), a residual stone in the pancreatic head, and a completely impacted mid-duct stone that could not be traversed with a conventional extraction balloon or basket.

SOP was performed using a pancreatoscope (an outer diameter of 3.2 mm) fitted with a transparent distal spacer cap ( Fig. 1a/b ). Under direct visualization, EHL (3.0-Fr EHL-probe) achieved complete stone fragmentation ( Fig. 2a/b ). Fragments were retrieved using a SOP basket (Boston Scientific, USA). Final pancreatography confirmed complete duct clearance with free contrast drainage, followed by the placement of a 10Fr/10cm pancreatic duct stent ( Video 1 ). The patient recovered without adverse events and was discharged symptom-free the following day.

**Fig. 1:**
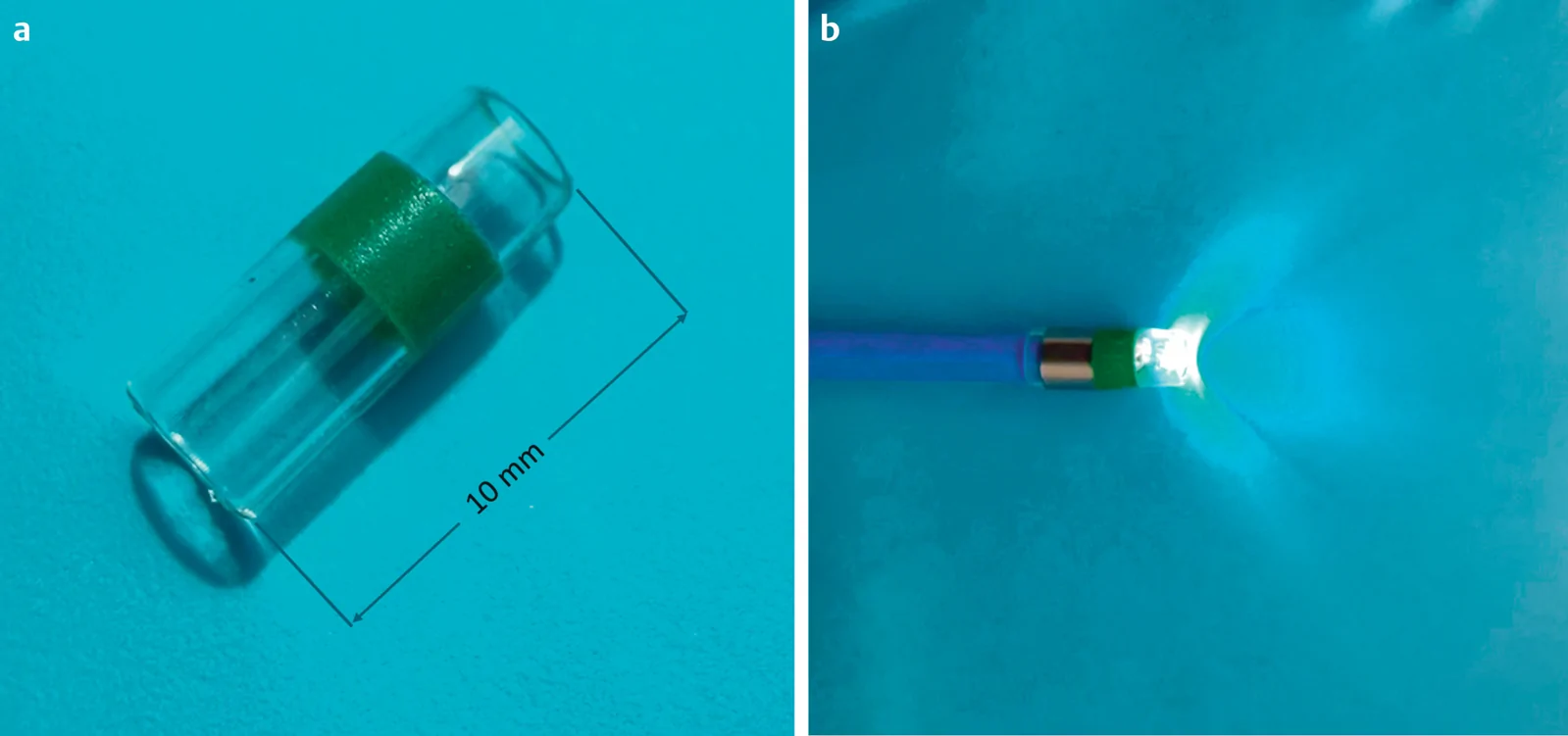
( **a** ) Transparent distal spacer cap. ( **b** ) Cap attached to the cholangioscope/pancreatoscope.

**Fig. 2:**
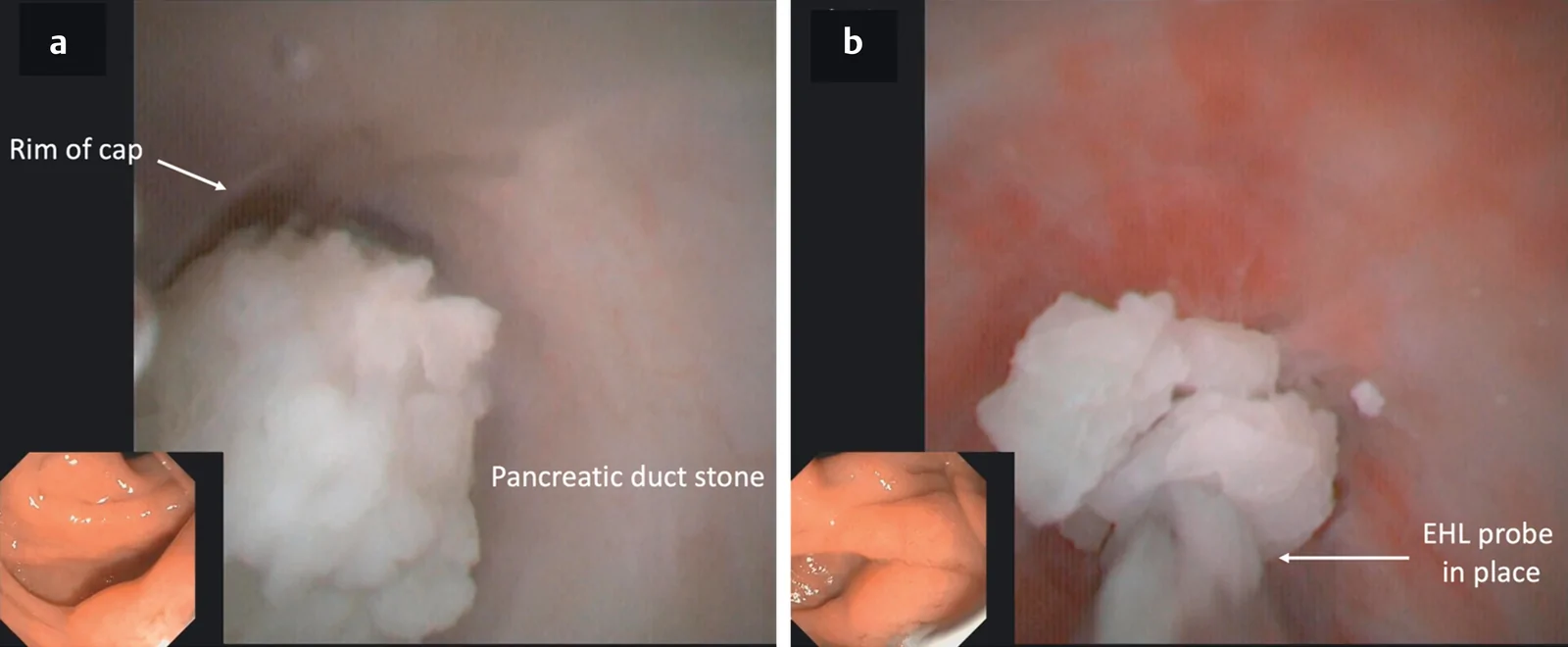
Cap-assisted single-operator pancreatoscopy with EHL of pancreatic duct stone: ( **a** ) cap-assisted visualization of stone and ( **b** ) the EHL probe in place. EHL, electrohydraulic lithotripsy.

**Video 1** Cap-assisted single-operator pancreatoscopy with electrohydraulic lithotripsy and complete extraction of impacted pancreatic duct stones in chronic calcifying pancreatitis.


Cap-assisted SOP is a simple, inexpensive technical refinement that improves papillary intubation, intraductal orientation, visualization, and centering of the EHL probe, enabling precise lithotripsy and complete ductal clearance after conventional techniques had failed. To our knowledge, this is the first report of cap-assisted SOP for pancreatic duct stone lithotripsy and may represent a valuable technical advance deserving prospective evaluation.

Endoscopy_UCTN_Code_TTT_1AR_2AL

## Declaration of generative artificial intelligence use

No generative artificial intelligence (AI) tools were used in the preparation of this manuscript or the associated figures, illustrations, clinical images, procedural video footage, or other submitted materials. Generative AI was also not used for writing, editing, or modifying the text. All contents were created, reviewed, and approved solely by the authors.
